# Relationship between serum total testosterone and prostate volume in aging men

**DOI:** 10.1038/s41598-021-93728-1

**Published:** 2021-07-08

**Authors:** Bo-Wen Xia, Si-Cong Zhao, Zong-Ping Chen, Chao Chen, Tian-Shu Liu, Fan Yang, Yong Yan

**Affiliations:** Department of Urology, Beijing Shijitan Hospital, Capital Medical University, 10th Tieyi Road, Haidian District, Beijing, 100038 China

**Keywords:** Urogenital diseases, Prostate

## Abstract

Total testosterone levels decline with age, while prostate volume and the prevalence of benign prostatic hyperplasia increase with age. We sought to investigate the correlation of serum testosterone levels with prostate volume in aging men. We analyzed clinical data obtained from 416 ostensibly healthy men who underwent routine health check-ups and recruited and collected data from these subjects 4 years later. We analyzed the correlation between prostate volume and relevant factors, as well as the correlation between changes in prostate volume and low testosterone over a 4-year period. Men with low testosterone had significantly larger prostate volume than those in the normal testosterone group (26.86 ± 8.75 vs. 24.06 ± 6.77 *P* = 0.02), and subjects with low testosterone had significantly higher levels of obesity-related factors, including waist circumference, body mass index, and insulin (all *P* < 0.001). After adjustment for age, testosterone level was negatively correlated with prostate volume (*P* = 0.004), and prostate volume and 4-year changes in prostate volume were associated with low testosterone. With increased testosterone level, prostate volume showed a significant linear decreasing trend. These findings provide evidence of the relationship between testosterone and prostate volume. Additional large studies are needed to confirm these preliminary results.

## Introduction

Benign prostate hyperplasia (BPH) is the most prevalent benign disease and main cause of lower urinary tract symptoms (LUTS) in aging men. BPH affects the quality of life of men by gradually leading to prostate volume (PV) enlargement and resulting in LUTS, recurrent urinary tract infections, acute urinary retention and other clinical symptoms^1^. Age is considered to be the most important risk factor for BPH, and the clinical symptom progression of BPH is also closely related to the increase in PV caused by advanced age^2^. Because of the high prevalence of BPH/LUTS in aging men and the high cost of treatment and early identification of patients at risk for progression events, it is essential to further identify risk factors for BPH/LUTS morbidity and progression^3^.

Studies have shown that testosterone is important for prostate growth and maintenance of functional integrity^4^. In recent years, androgen deficiency in aging men, later renamed late-onset hypogonadism, has become a frequent topic of discussion among andrologists, dermatologists, endocrinologists, and urologists^5^. Testosterone has important effects on cardiovascular and liver disease as well as mental health, and androgen deficiency may adversely affect organ system function and lead to severe quality of life impairment^6,7^. The incidence of testosterone deficiency is estimated at approximately 20% among men over the age of 60, 30% over 70 and 50% over 80^8^. A study suggested that, independent of multiple risk factors and several preexisting health conditions, low levels of circulating total testosterone are associated with a 40% increase in mortality in older men^9^.

Although testosterone is closely related to PV, both change significantly with age, but how testosterone affects PV is unclear. Partly based on human cross-sectional^10–12,14,15,33,34^ and longitudinal studies^13,16^ researchers have found that elevated testosterone levels increase PV, while others have found no or negative correlation between prostate volume and testosterone. Thus, in older men, the effects of androgens on PV and LUTS remain controversial. Current studies have found that androgen replacement therapy (TRT) can relieve LUTS symptoms^17^, and high serum testosterone levels are associated with reduced risk of BPH^18^. Therefore, it has been suggested that appropriate testosterone may reduce the inflammatory response in the prostate^19^.

Some research has yielded results, but more systematic studies are needed to clarify the relationship between testosterone and PV in aging men. In addition, few studies have looked at the correlation between androgen and PV in Asian populations, and there is also an effect of race on androgen levels^13,14^. Therefore, in the present study, we aimed to explore the relationship between serum testosterone levels and several related factors, especially PV, in aging Asian men by collecting data from healthy Chinese people.

## Methods

### Study subjects

**Figure 1:**
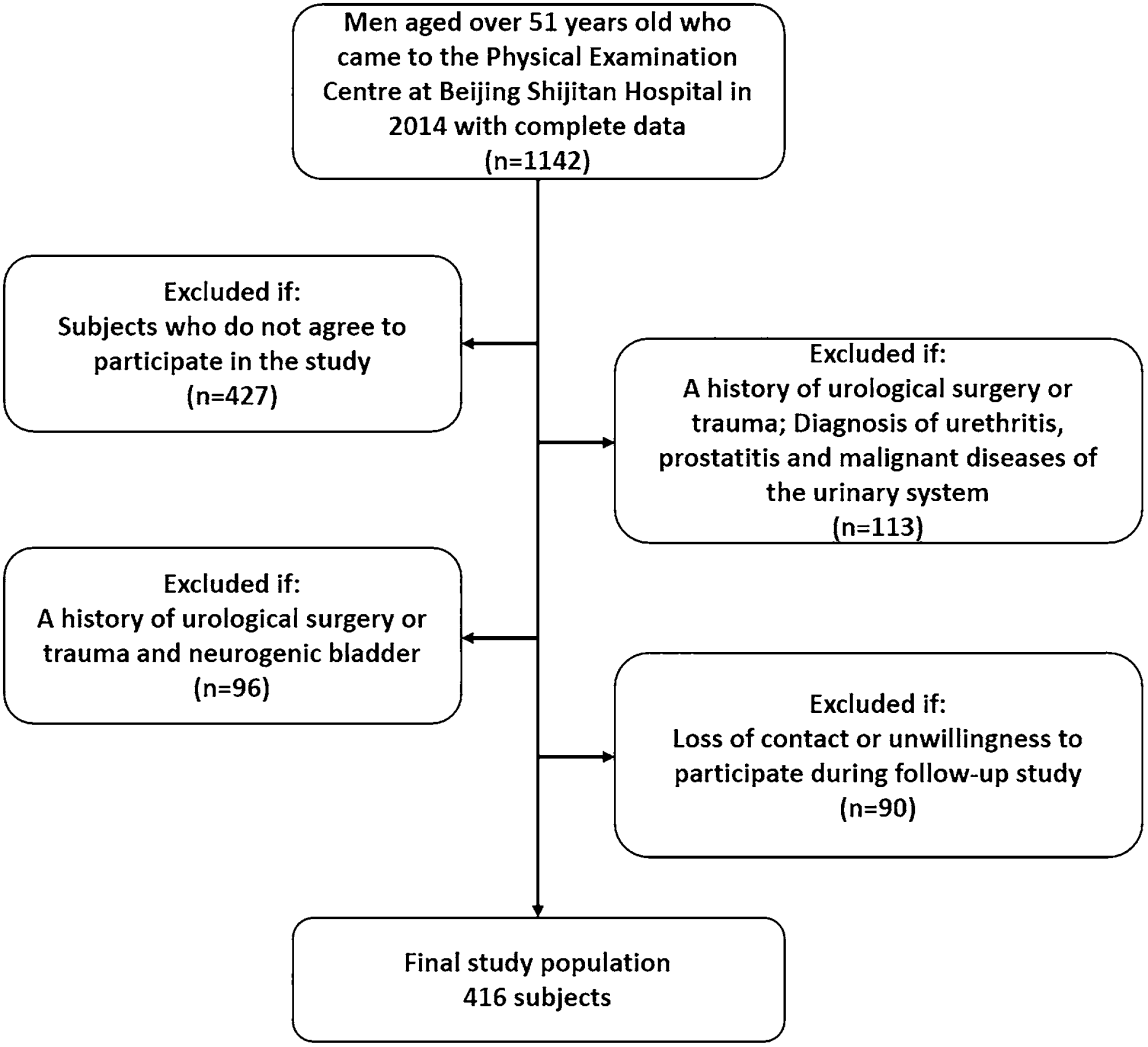
Flow chart of selection of the included subjects.

All subjects were recruited consecutively by routine physical examination. After excluding those who met the exclusion criteria and who did not agree to participate in our research, 416 subjects aged over 51 years in the Beijing area participated in the present study by two routine physical examination programs from October 2014 and October 2018 (Fig. 1). Only men who completed the follow-up were included in the analysis. Our research used the following exclusion criteria for both data collection periods: (1) urethritis and prostatitis, urethral stricture, or neurogenic bladder; (2) a history of urological surgery or trauma; (3) diagnosis of malignant diseases of the urinary system; and (4) neurological diseases that may affect urinary function. These study protocols were approved by the ethics committee of Beijing Shijitan Hospital affiliated with Capital Medical University and China Railway Corporation, and all subjects volunteered to participate in the experiment after providing informed consent and agreeing in writing prior to registration. All experiments were performed in accordance with relevant guidelines and regulations.

### Data collection

All subjects (including those enrolled in October 2014 and October 2018) completed the International Prostate Symptom Sore (I-PSS) questionnaire after Chinese translation^20^, and anthropometric measurements, including waist circumference (cm), blood pressure (mmHg), body weight (kg) and height (cm), were measured by trained nurses using a standardized protocol. Body mass index (BMI) was calculated by using the formula (weight/height squared), and PV (cm3) was measured by suprapubic ultrasonography (3.5 MHz; Hitachi EUB-400, Tokyo, Japan) using the formula for an elliptic volume (height × width × length × π/6); the maximum urinary flow rate (Qmax) was determined by uroflowmetry at a voided volume of > 150 ml.

All blood measurements were performed using fresh serum obtained after a 12-h fast to minimize the confounding effects of diurnal variation on hormone concentrations. The measured parameters included prostate-specific antigen (PSA), fasting blood glucose (FBG), triglycerides, high-density lipoprotein cholesterol (HDL-C), and low-density lipoprotein cholesterol (LDL-C). Insulin, 5α-dihydrotestosterone (DHT), sex hormone-binding globulin (SHBG), total testosterone, leptin, resistin, adiponectin, IL-6, C-reactive protein (CRP) and TNF-α were measured using enzyme-linked immunosorbent assay methods. The homeostasis model assessment of insulin resistance (IR) index was calculated using the HOMA algorithm: glucose (mg/dL) × insulin (μU/mL)/405^21^. Blood biochemical tests were collected only from the 416 subjects recruited from October 2014 to August 2015 and were tested in a single batch to reduce variability.

### Statistical analysis

We defined a low testosterone level as < 300 ng/dL. Clinical characteristics were compared between subjects with and without low testosterone comparatively by using independent t-tests for continuous variables and chi-square tests for categorical variables. The Shapiro-Smirnov test was used to verify whether the data fit a normal distribution, all continuous variables that did not conform to the normal distribution were analyzed according to the distribution, and all non-normal variables conformed to the normalized data after transformation. Age-adjusted linear regression models were used to evaluate the associations of related factors with PV values. Logistic regression models adjusted for only age or age and insulin were used to assess associations of low testosterone and PV levels and to calculate odds ratios (ORs). Furthermore, we divided subjects into four groups based on the quartiles of serum levels of testosterone to investigate whether there was a linear difference in PV values among the groups by using ANOVA. The data were analyzed using SPSS software version 13.0 for Windows (SPSS Inc., Chicago, IL, USA), and two-tailed *P* values < 0.05 indicated statistical significance.

## Results

The baseline characteristics of the subjects are presented in Table 1. The mean age in the study sample was 69.3 ± 8.31 years, and the mean TT level was 4.61 ± 1.68 ng/ml. The mean IPSS of the study population was 12.98 of 35, and the mean PV was 24.47 cm3. BMI, waist circumference, PV, MetS, PV, insulin, HOMA IR, DHT, and SHBG, levels were significantly different between subjects in the low testosterone and normal testosterone groups (all *P* < 0.05). In addition, subjects in the lower TT group were significantly more obese (BMI: 26.40 ± 3.34 vs. 24.47 ± 3.01 *P* < 0.001; waist circumference: 91.75 ± 8.87 vs. 85.95 ± 8.38 *P* < 0.001) and had larger PV (26.86 ± 8.75 vs. 24.06 ± 6.77 *P* = 0.02) than those in the control group.

**Table 1:** Baseline characteristics.

Characteristics	Overall (416)	Low Testosterone(61)	Normal Testosterone(355)	*P*-value
Age	68.99 ± 8.18	69.15 ± 7.99	68.00 ± 9.20	0.359
BMI, kg/m2	24.76 ± 3.13	26.40 ± 3.34	24.47 ± 3.01	** < 0.001**
Waist circumference, cm	86.80 ± 8.69	91.75 ± 8.87	85.95 ± 8.38	** < 0.001**
SBP, mmHg	137.13 ± 15.66	138.39 ± 15.47	136.91 ± 15.71	0.494
DBP, mmHg	79.17 ± 10.51	79.84 ± 11.55	79.06 ± 10.33	0.593
Elevated blood pressure, *n* (%)	70.4%	72.1%	70.1%	0.879
FBG, mg/dL	102 ± 26.01	99.42 ± 22.13	103.33 ± 26.60	0.278
Triglycerides, mg/dL	162.37 ± 146.97	151.64 ± 92.35	164.22 ± 154.44	0.538
HDL-C, mg/dL	47.31 ± 11.24	46.51 ± 13.69	47.46 ± 10.79	0.545
LDL-C, mg/dL	111.42 ± 30.52	115.06 ± 35.52	110.79 ± 29.59	0.378
Total cholesterol, mg/dL	187.69 ± 37.67	190.49 ± 42.57	187.21 ± 26.80	0.571
PV, cm^3^	24.47 ± 7.15	26.86 ± 8.75	24.06 ± 6.77	**0.020**
Q_max_, mL/s	15.60 ± 6.12	15.15 ± 5.99	15.68 ± 6.14	0.535
PSA, ng/mL	1.39 ± 1.16	1.48 ± 1.44	1.38 ± 1.11	0.545
IPSS	12.98 ± 7.65	13.36 ± 7.86	12.92 ± 7.61	0.677
Urination symptom score	8.68 ± 5.10	8.95 ± 5.24	8.64 ± 5.08	0.657
Emptying symptom score	4.30 ± 2.57	4.41 ± 264	4.28 ± 2.56	0.719
Quality of Life score	2.87 ± 1.60	2.98 ± 1.64	2.85 ± 1.59	0.559
Insulin, pmol/L	51.31 ± 31.09	66.38 ± 38.64	48.72 ± 28.88	**0.001**
HOMA IR	2.13 ± 1.34	2.65 ± 1.52	2.04 ± 1.29	**0.004**
DHT, pg/mL	381.84 ± 238.25	393.29 ± 248.94	315.22 ± 147.27	**0.001**
SHBG, nmol/L	70.85 ± 35.89	46.27 ± 27.71	75.08 ± 35.47	** < 0.001**
Testosterone, ng/mL	4.61 ± 1.68	2.56 ± 0.48	4.97 ± 1.56	** < 0.001**

*P* were calculated by independent t-test† and chi-squared test‡

Bold indicates statistically significant values.

The total number of subjects was 416.

*BMI* body mass index, *SBP* systolic blood pressure, *DBP* diastolic blood pressure, *FBG* fasting blood glucose, *HDL-C* high-density lipoprotein cholesterol, *LDL-C* low-density lipoprotein cholesterol, *PV* prostate volume, *Qmax* the maximum urinary flow rate, *PSA* prostate-specific antigen, *IPSS* International Prostate Symptom Sore, *DHT* 5α-dihydrotestosterone, *SHBG* sex hormone binding globulin, *FAI*% free androgen index.

The results of the age-adjusted linear regression models shown in Table 2 indicated that BMI, waist circumference, blood pressure (including systolic pressure and diastolic pressure), insulin, HOMA IR, SHBG and TT were significantly correlated with PV levels (all *P* < 0.05). Among the indicators related to obesity, BMI, waist circumference, insulin and IR were positively correlated with PV levels, while serum TT levels and SHBG were negatively associated with PV levels (*P* = 0.004 and *P* = 0.042, respectively). There was no significant correlation between DHT, estradiol and PV levels.

**Table 2:** Age-adjusted regression analysis of factors that affect PV levels.

Characteristics	B	*t*	95% CI	*†P*-value
BMI, kg/m2	0.322	6.620	0.226, 0.417	** < 0.001**
Waist circumference, cm	0.312	6.610	0.222, 0.411	** < 0.001**
SBP, mmHg	0.215	4.278	0.116, 0.314	** < 0.001**
DBP, mmHg	0.120	2.399	0.022, 0.218	**0.017**
FBG, mg/dL	− 0.017	− 0.349	− 0.114, 0.079	0.727
Triglycerides, mg/dL	0.068	1.382	− 0.029, 0.166	0.168
HDL-C, mg/dL	− 0.063	− 1.287	− 0.160, 0.033	0.199
LDL-C, mg/dL	− 0.039	− 0.793	− 0.135, 0.057	0.428
Total cholesterol, mg/dL	− 0.077	− 1.578	− 0.173, 0.019	0.115
Insulin, pmol/L	0.155	3.102	0.057, 0.253	**0.002**
HOMA IR	0.140	2.839	0.043, 0.237	**0.005**
DHT, pg/mL	− 0.056	− 1.137	− 0.153, 0.041	0.256
SHBG, nmol/L	− 0.107	− 2.044	− 0.209, − 0.004	**0.042**
Testosterone, ng/mL	− 0.141	− 2.886	− 0.237, − 0.045	**0.004**
FAI %	− 0.004	− 0.070	− 0.106, 0.099	0.944

Bold indicates statistically significant values.

*CI* confidence interval, *PV* prostate volume, *BMI* body mass index, *SBP* systolic blood pressure, *DBP* diastolic blood pressure, *FBG* fasting blood glucose, *HDL-C* high-density lipoprotein cholesterol, *LDL-C* low-density lipoprotein cholesterol, *DHT* 5α-dihydrotestosterone, *SHBG* sex hormone binding globulin, *FAI*%, free androgen index.

^†^*P*-value were calculated according to multivariate linear regression analyses after adjustment of age.

As shown in Table 3, there was a significant association of PV with low testosterone after adjusting for age (OR = 1.052 *P* = 0.004). Low testosterone was also correlated with PV values after 4 years of follow-up (OR = 1.056 *P* = 0.002), and the PV growth of the low testosterone group was significantly greater than that of the normal testosterone group after 4 years (OR = 3.108 *P* < 0.001). However, after adjusting for age, BMI, and insulin, only the PV value difference over the 4-year period was significantly associated with low testosterone (OR = 2.642 *P* < 0.001).

**Table 3:** Associations of Low testosterone and PV levels after 4 years.

Characteristics	OR	95% CI	*P*-value
**Adjustment for Age**			
PV cm^3^	1.052	1.016, 1.090	**0.004**
PV-4 year after cm^3^	1.056	1.021, 1.093	**0.002**
*PV value difference cm^3^	3.108	1.838, 5.255	** < 0.001**
**Adjustment for Age, BMI, Insulin**			
PV cm^3^	1.027	0.990, 1.066	0.152
PV-4 year after cm^3^	1.031	1.000, 1.017	0.096
*PV value difference cm^3^	2.642	1.539, 4.536	** < 0.001**

*PV value difference, PV-4 year after subtract PV.

**Figure 2:**
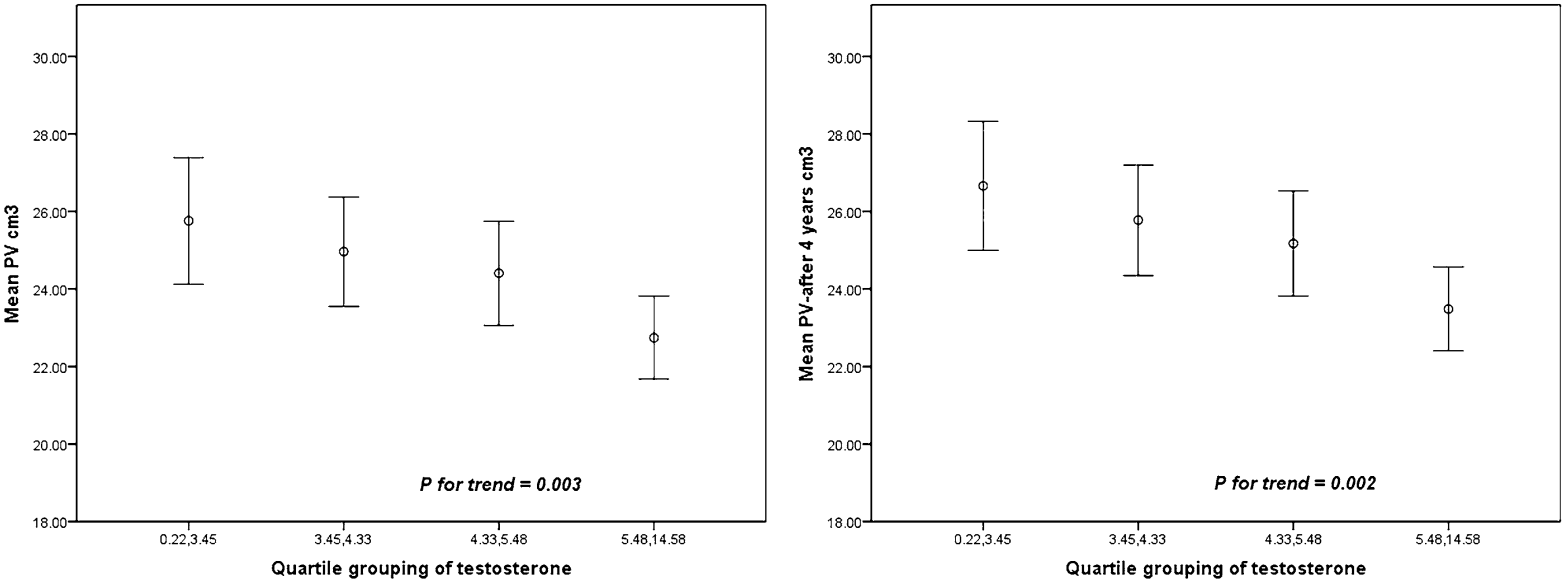
Mean prostate volume and prostate volume after 4 years adjusted for age. White circle indicates mean. Lower and upper bar indicates 95% CI.

Finally, we divided subjects into four groups based on the quartile serum testosterone levels to investigate whether there was a trend deviation of PV levels among the groups (Fig. 2). After adjusting for age, the mean PV and PV after 4 years significantly and gradually decreased as serum testosterone levels increased (*P* = 0.003 and *P* = 0.002, respectively).

## Discussion

The association between total testosterone levels and prostate volume has been controversial. The results of the present study show that TT level was negatively correlated with PV after adjusting for age in Chinese aging men. Meanwhile, the rate of PV growth in aging patients with low testosterone was significantly greater than the normal TT level group after adjusting for age.

As an increasing number of countries have aging societies, scientists are concerned about symptoms in older men associated with changes in sex hormones, particularly changes in testosterone levels^22^. Testosterone levels decrease with age, a syndrome defined by the ISSAM (International Society for the Study of the Aging Male) as androgen deficiency or late-onset hypogonadism, which can cause a number of physical complaints, including decreased libido, erectile quality, and intellectual activity; fatigue; depression; and irritability^23^. An observational cross-sectional study of testosterone and age found that TT and free testosterone decreased with age (0.4%/year and 1.2%/year)^24^. Feldman, H A also found that serum testosterone levels declined steadily after age 40, with an average annual decrease of 0.8% in TT. Furthermore, deteriorating health may accelerate age-related declines in testosterone levels based on longitudinal data^22^. Our results showed that subjects in the low androgen group were older than those in the control group, but there was no significant difference. This may have been related to the androgen grouping. In the present study, there was an association between age and TT (*P* = 0.002).

Obesity was also associated with decreased TT levels in the prospective prevalence study of Calderon, B et al.^25^, which determined that 45% of moderately to severely obese patients had low testosterone levels, and serum TT levels were negatively correlated with blood glucose and insulin resistance. Studies have also found that the proportion of subnormal free testosterone in obese subjects is significantly higher than that in normal and lean people, and free testosterone was negatively correlated with BMI (r = − 0.18 *P* < 0.001) and significantly lower in diabetic patients. Our results also showed that subjects in the low testosterone group had significantly higher waist circumference and BMI, as well as higher insulin levels and HOMA IR, than those in the normal testosterone group.

In contrast to the relationship between TT and age, the prevalence of BPH increased significantly with age^2^. Some retrospective studies have also shown that there is a positive correlation between age and PV; meanwhile, the fastest increasing rate of PV was between the ages of 50 and 69^1,26^. In our study, we found a significant correlation between prostate volume and age (*P* = 0.030, data not shown). As with TT, PV was also associated with obesity and insulin, and Vignozzi, L et al. found that obesity and insulin could have a detrimental effect on the prostate and are a risk factor for BPH progression^27^. A similar study indicated that PV significantly correlated with fasting serum insulin (*P* = 0.028). In our study, there was a positive correlation between PV with insulin and insulin resistance. Thus, age and obesity-related factors are both correlated with TT and PV.

Some studies have looked at the relationship between serum androgen levels in men and clinical prostatic hyperplasia or prostate volume, but the results have been inconsistent. Meikle et al.^15^ found that PV was negatively correlated with age-adjusted serum TT, DHT, and SHBG levels in 214 male twins between 25 and 75 years old. Another study came to a similar conclusion in Asians; with decreased TT, the IPSS score and PV all significantly increased^10^. Roberts et al. indicated that PV was negatively associated with bioavailable testosterone level (rs = − 0.13, *P* < 0.05). However, after adjusting for age, the results were not statistically significant^13^. In contrast, Nukui M et al.^35^ found that PV was positively correlated with TT, but this finding only existed in groups with PV > 25 ml in a cross-sectional study of 226 subjects. Other studies have also failed to find a significant association between TT level and PV^12,14^.

In fact, both TT and PV are correlated with race and age, and the heterogeneity of different studies may arise from the differences in age and region of the subjects. Our present results suggest that there is a significant negative association between PV and TT levels after age adjustment, and subjects with low testosterone levels had significantly larger prostate volumes than men with normal testosterone levels after 4 years. After adjusting for age, BMI and insulin levels, there was no statistical association between PV and TT levels; however, there was still a statistical correlation between increased PV over 4 years and TT levels (OR = 2.642 *P* < 0.001). Similar to our current findings, a cross-sectional study of 406 Australians found a negative correlation between age and serum testosterone levels (r = − 0.265; *P* < 0.001), and BMI was inversely correlated to TT (r = − 0.42; *P* < 0.001). After adjusting for age and sampling time, PV measured by transrectal ultrasound was negatively correlated with TT, free testosterone, and bioavailable testosterone^36^.

TRT is increasingly used in older men. A study of 13 hypogonadal men aged 25 to 35 who underwent TRT found a significant increase in PV (*P* < 0.001)^27^. Other longitudinal researchers have shown that testosterone supplementation increases PV by an average of 12%^29^. However, Morales^30^ found that there was no significant difference in PV between men treated with testosterone and those treated with placebo. A well-controlled RCT study of 44 men with hypogonadism showed no significant increase in testosterone levels in prostate tissue and little change in treatment-related prostate volume in men treated with TRT, despite significantly increased serum TT levels^31^. Long-term testosterone therapy in hypogonadal men showed significant improvements in urinary function and QoL, and PV was also correlated with testosterone treated^32^. In a similar study, testosterone-treated group showed a smaller increase in PV after eight years than control group^33^. It is possible that TRT may affect normal or low gonadal function in men with primary hypogonadism, but there is a lack of confirmation in men with aging hypogonadism. In the present study, increased serum TT and PV levels showed a linear trend of significant decrease.

Among sex hormones other than TT, Schatzl et al.^34^ found that estradiol (but not testosterone) was associated with PV (r = 0.17, *P* = 0.01). Joseph et al.^14^ found that SHBG and endogenous steroid hormones were correlated with prostate volume, with SHBG being negatively correlated. Our study also found a negative correlation between SHBG and PV (*P* = 0.042). In older men, however, the relationship between sex steroids and PV is more complex than a single effect. Further systematic studies are needed to analyze the correlation between all sex hormones and PV.

This article has some shortcomings that need to be noted. Firstly, the study was implemented in a single institution setting, which may be subject to selection bias and is not fully representative of the overall population. Further scaling is needed to validate our results. Second, the current cross-sectional study cannot reach an accurate conclusion and can only provide some evidence for the follow-up study, which requires further longitudinal and prospective studies. Third, compared with mass spectrometry-based measurements, immunoassay may not be the most accurate method when serum testosterone levels are low due to the introduction of random noise into the models. Measurement errors should be reduced as much as possible by professional and skilled laboratory personnel. A further limitation was that subjects in the present study were older on average, which may have led to some other complications of aging that affect PV, such as diabetes or metabolic syndrome. The effects of these age-related diseases on the prostate are very complex and difficult to fully correct, and the mechanism by which androgen deficiency in aging men influences PV should be further studied.

## Conclusion

The results of our study suggest that testosterone levels are negatively correlated with prostate volume, and BMI, waist circumference, and insulin are negatively correlated with PV in older patients. In addition, men with low testosterone developed a larger prostate than men with normal testosterone. These findings will contribute to a better understanding of the role of TT in LUTS/BPH.
